# Profound Hyperkalemia and Anemia in a Dialysis Patient With a Gastrointestinal Bleed

**DOI:** 10.7759/cureus.34437

**Published:** 2023-01-31

**Authors:** Sara Biladeau, Ryan Grell

**Affiliations:** 1 Anesthesiology and Perioperative Medicine, University of Louisville School of Medicine, Louisville, USA

**Keywords:** perioperative medicine, preoperative evaluation, hyperkalemia induced ekg changes, gi anesthesia, perioperative anesthesia service, anesthesia for upper and lower gi, on dialysis, massive lower gi bleeding, severe anemia, severe hyperkalemia

## Abstract

An 80-year-old male receiving dialysis three times per week presented to the emergency room with general malaise after missing four consecutive dialysis appointments. During his workup, he was noted to have a potassium of 9.1 mmol/L, hemoglobin of 4.1 g/dL, and an electrocardiogram showing a first-degree atrioventricular (AV) block, a right bundle branch block, peaked T waves, and a wide QRS complex. During emergent dialysis and resuscitation, the patient suffered respiratory failure and was intubated. The next morning, he underwent an esophagogastroduodenoscopy (EGD), which found a healing duodenal ulcer. He was extubated the same day and was discharged in stable condition a few days later. This case appears to report the highest observed potassium coupled with significant anemia in a patient not affected by cardiac arrest.

## Introduction

Chronic anemia and mild hyperkalemia in patients with chronic kidney disease are not uncommon; however, profound hyperkalemia is rare. As with other cases of hyperkalemia, the temporizing and definitive treatments are the same; however, the urgency and potential sequelae of delaying immediate treatment are quite severe [1]. The addition of severe anemia and the need for an urgent esophagogastroduodenoscopy (EGD) to manage a potentially severe gastrointestinal bleed further complicated this case and added an element of uncertainty as to which procedure - dialysis or EGD - should be prioritized and completed first.

## Case presentation

An 80-year-old male with a history of hypertension, chronic obstructive pulmonary disease (COPD) on 2 L of home oxygen, and end-stage renal disease requiring hemodialysis three times per week presented to the emergency department with a chief complaint of malaise. Of note, he reported that he missed his last four sessions of hemodialysis and developed hematochezia, with clots accompanying his bowel movements for the past three days.

On physical examination, he was alert, oriented, and in no acute distress. He was noted to be in sinus rhythm with a blood pressure of 180/62, heart rate of 80 beats per minute, respiratory rate of 20 breaths per minute, oxygen saturation of 96% on room air, and a temperature of 100.1 °F. His laboratory results were notable for hemoglobin of 4.1 g/dL (normal reference range 13-17.5 g/dL), potassium of 9.1 mmol/L (normal reference range 3.7-5 mmol/L), troponin I of 49 ng/L (normal reference range <19 ng/L), and brain natriuretic peptide of 1,118 pg/mL (normal reference range 5-100 pg/mL; Table 1).

**Table 1:** Preintervention laboratory results.

Laboratory test	Result	Laboratory test	Result
Troponin I	49 ng/L	Sodium	140 mmol/L
Brain natriuretic peptide	1,118 pg/mL	Potassium	9.1 mmol/L
White blood cell count	11.8 × 10^3^ mm^-3^	Chloride	104 mmol/L
Hemoglobin	4.1 g/dL	Carbon dioxide	18.1 mmol/L
Hematocrit	12.1	Blood urea nitrogen	185 mg/dL
Platelet count	244 × 10^3^ mm^-3^	Creatine	17.49 mg/dL
Lactic acid level	1.1 mmol/L	Glucose	108 mg/dL

**Figure 1:**
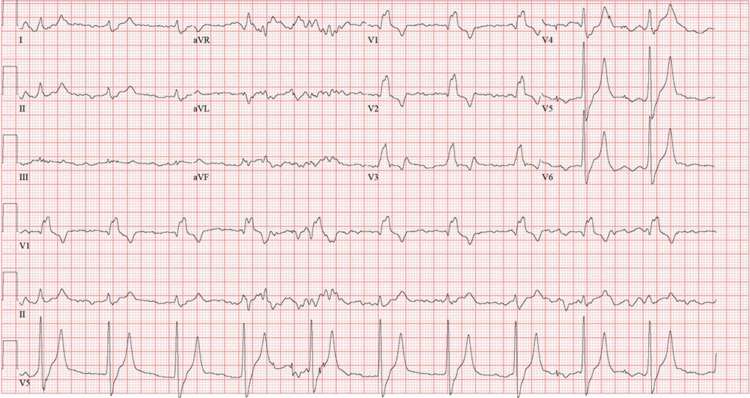
Preintervention electrocardiogram.

His electrocardiogram (EKG) revealed a first-degree AV block, right bundle branch block, peaked T waves, and wide QRS complex (Figure 1).

The emergency medicine team administered 1 g intravenous (IV) calcium gluconate, 10 units IV regular insulin, 25 g IV dextrose, and 50 mEq IV sodium bicarbonate. They ordered a blood type and crossmatch for three units of packed red blood cells, which revealed significant antibodies, which would delay the availability of blood products for one to two hours. The nephrology and gastroenterology services were consulted for workup and management recommendations.

Our anesthesiology service was consulted by the gastroenterology service for an anesthesia preoperative assessment and optimization recommendations for an urgent EGD to determine the etiology of the patient's gastrointestinal bleed. Due to the patient’s profound hyperkalemia, the anticipated increase in potassium with the administration of packed red blood cells, the anticipated delay in receiving crossmatched blood products, and the relative stability of the patient, we requested the patient be transferred first to the dialysis unit for emergent hemodialysis and eventual administration of packed red blood cells.

**Figure 2:**
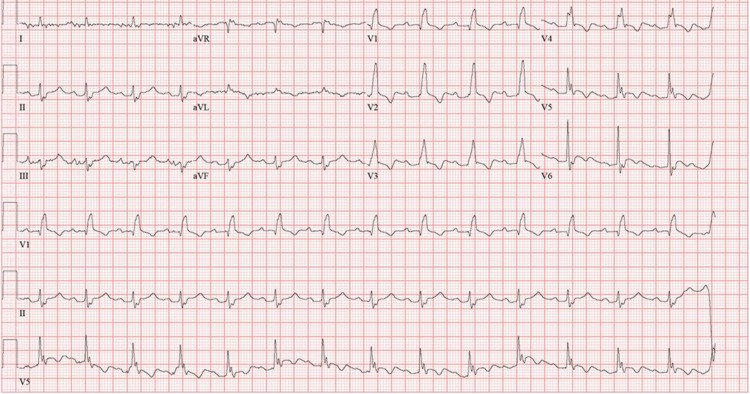
Postintervention electrocardiogram.

Shortly after the initiation of dialysis, the patient developed increasing shortness of breath accompanied by decreasing SpO2 to 82% and was intubated by the rapid response team with etomidate and rocuronium for acute hypoxic respiratory failure due to presumed fluid overload. After dialysis, the patient received three units of packed red blood cells and improved his hemoglobin to 6.5 g/dL. His postdialysis potassium was 4.6 mmol/L. After remaining mechanically ventilated overnight, he was transfused with one additional unit of packed red blood cells, with a resultant hemoglobin of 8.2 g/dL. His repeat EKG showed considerable improvement with the disappearance of the right bundle branch block and resolution of peaked T waves (Figure 2).

The next morning, the patient underwent an EGD, which revealed a 1-cm-long and 3-mm-wide duodenal ulcer, which was in the early stages of healing. He was extubated postprocedure and monitored with serial complete blood counts for two days before being discharged home in stable condition, with a prescription for twice daily proton pump inhibitors and a recommendation for the avoidance of nonsteroidal anti-inflammatory agents.

## Discussion

An excess of extracellular potassium can lead to a variety of clinical manifestations that can be seen upon initial evaluation, which can aid in the diagnosis of hyperkalemia. Both skeletal and cardiac muscle cells depend upon the sodium-potassium ATPase pump to maintain a negative intracellular resting potential by using adenosine triphosphate (ATP) to influx potassium and efflux sodium. In patients with hyperkalemia, the resting membrane potential is less negative and closer to the threshold for an action potential to cause muscle contraction. Initially, this can cause excitation, but, over time, the increase in inactivated sodium channels leads to decreased excitability as well as slowing of electrical impulses, leading to severe weakness or even paralysis of skeletal muscles [2,3].

The effects of hyperkalemia on cardiac muscles include conduction abnormalities and EKG changes. Although the correlation with the serum potassium can be poor, the classic EKG changes in hyperkalemia include peaked T waves, shortened QT interval, lengthened PR and QRS interval, flattened P waves, and widened QRS complex that can ultimately lead to a sine wave pattern. The first two changes are due to the increased activation of the rapid-acting delayed rectifier potassium channel *I*_Kr_. The latter changes are due to prolonged depolarization and slowed conduction/longer action potential through the myocardium. Other conductance abnormalities that can be seen include atrioventricular nodal blocks, apparent bundle branch blocks due to intraventricular conduction delay, sinoventricular rhythms due to ventricular activity without atrial activity, and ventricular fibrillation after nodal conduction ceases and junctional rhythms appear [2,3].

Additionally, metabolic acidosis can be seen from decreased ammonium excretion in the setting of hyperkalemia. An intracellular alkalosis is seen using the sodium-hydrogen pump shunting potassium at the expense of hydrogen ion efflux, which, in turn, decreases ammonium excretion and bicarbonate reabsorption. In addition, there is decreased ammonium reabsorption in the thick ascending loop of Henle as the potassium competes with the ammonium and leads to a decreased recycling of ammonium that is ultimately secreted in the collecting duct, leading to a metabolic acidosis [4].

Hyperkalemia is a potentially life-threatening condition because of the profound adverse cardiac events seen at very high levels of potassium. A variety of temporary and definitive techniques can be used to decrease serum potassium. Temporary solutions include strategies, which activate the sodium-potassium ATPase to shift potassium inside cells (e.g., insulin or beta agonists like albuterol) [5] and decrease blood acidity during metabolic acidosis to stimulate a concurrent transporter to shift potassium into cells (e.g., sodium bicarbonate) [6,7]. Definitive techniques include removing potassium from the body entirely through excretion in urine (diuretics such as furosemide), fecal matter (potassium binders), or blood filtration (hemodialysis) [6,8]. In addition, temporary stabilization of the cardiac myocytes during this process is also warranted before or during hyperkalemia treatment. Calcium salts, such as calcium gluconate or calcium chloride, antagonize the effects of potassium on cardiac myocytes and can help to delay the progression of cardiac symptomatology [5].

Equally important to the treatment of hyperkalemia is the avoidance of medications and situations that can increase the extracellular potassium concentration. The commonly utilized neuromuscular blocking agent succinylcholine can transiently raise serum potassium levels by 0.5 to 1.0 mmol/L in healthy patients and up to 2 mmol/L in patients with burn injuries, end-stage renal disease, and certain neuromuscular diseases. Beta-adrenergic receptor blockers (e.g., metoprolol), potassium-sparing diuretics (e.g., spironolactone), metabolic acidosis, ischemic necrosis, hemolysis, rhabdomyolysis, and severe hyperglycemia have all been found to increase serum potassium as well. While it is not always possible to avoid these medications and situations, it is essential to do whenever clinically feasible to lower the likelihood of inducing significant hyperkalemia in susceptible patients [9].

## Conclusions

Profound hyperkalemia and anemia are both potentially life-threatening conditions. In this case, we prioritized definitively treating the patient’s hyperkalemia first because he displayed significant EKG changes, hemodynamic stability, and significant red blood cell antibodies that would delay transfusion. Additionally, the risk of sudden cardiac collapse in the setting of life-threatening hyperkalemia was also quite high. Ultimately, we successfully treated this patient’s hyperkalemia and anemia, and he was discharged in stable condition shortly afterward. As with all patients with multiple life-threatening conditions, care should be taken to personalize the prioritization of treatments based on individual risk and clinical circumstances.
